# Recent advances in allylation of chiral secondary alkylcopper species

**DOI:** 10.3762/bjoc.21.51

**Published:** 2025-03-20

**Authors:** Minjae Kim, Gwanggyun Kim, Doyoon Kim, Jun Hee Lee, Seung Hwan Cho

**Affiliations:** 1 Department of Chemistry, Pohang University of Science and Technology (POSTECH), Pohang, 37673, Republic of Korea; 2 Department of Advanced Materials Chemistry, Dongguk University WISE, Gyeongju 38066, Republic of Korea

**Keywords:** allylic substitution, chiral secondary organocopper, copper-mediated reaction, stereoselectivity

## Abstract

The transition-metal-catalyzed asymmetric allylic substitution represents a pivotal methodology in organic synthesis, providing remarkable versatility for complex molecule construction. Particularly, the generation and utilization of chiral secondary alkylcopper species have received considerable attention due to their unique properties in stereoselective allylic substitution. This review highlights recent advances in copper-catalyzed asymmetric allylic substitution reactions with chiral secondary alkylcopper species, encompassing several key strategies for their generation: stereospecific transmetalation of organolithium and organoboron compounds, copper hydride catalysis, and enantiotopic-group-selective transformations of 1,1-diborylalkanes. Detailed mechanistic insights into stereochemical control and current challenges in this field are also discussed.

## Introduction

The transition-metal-catalyzed regio- and enantioselective allylic substitution represents a pivotal methodology in organic synthesis, providing remarkable versatility for complex molecule construction [1–4]. The significance of this transformation lies in its unique ability to efficiently create a stereogenic center while forming new carbon–carbon or carbon–heteroatom bonds (e.g., C–N, C–O, and C–S) with excellent selectivities.

**Scheme 1:**
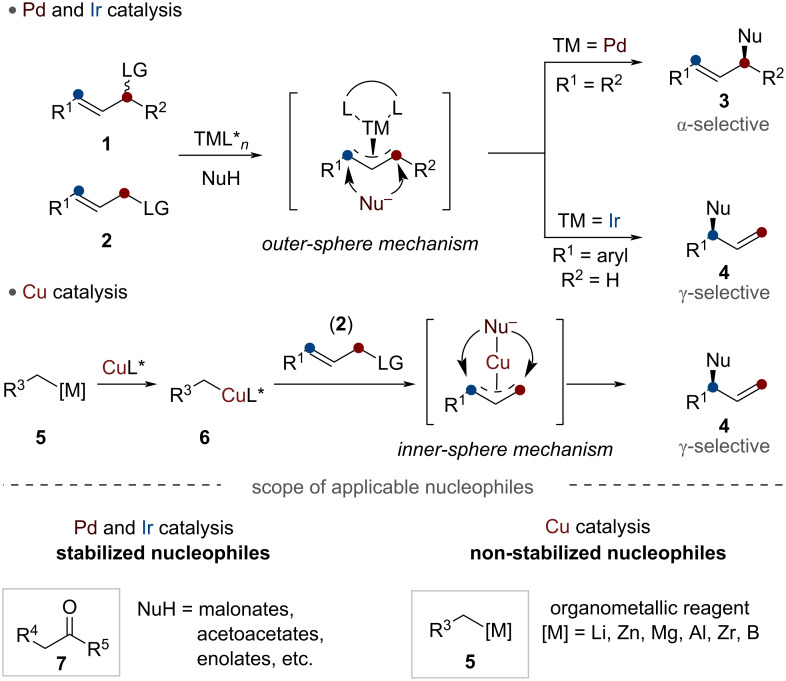
Representative transition-metal catalysis for allylic substitution.

The field of metal-catalyzed allylic substitution has evolved significantly since its inception (Scheme 1). Early studies were mainly focused on palladium catalysts [5–8], as demonstrated by the independent pioneering works of Tsuji and Trost in the 1960s and 1970s, respectively. While palladium catalysts demonstrated excellent reactivity with soft stabilized nucleophiles in the eponymous Tsuji–Trost reaction, they faced a significant limitation: poor regioselectivity with non-symmetrical allylic substrates **2**. This constraint led to the predominant development of Pd-catalyzed methods using symmetric 1,3-disubstituted allylic substrates **1** that contain a leaving group in the allylic terminus.

In search of complementary approaches, other transition metals including W [9], Mo [10], Ru [11–12], Rh [13–15], and Ni [16–17] have been explored. Among these alternatives, Ir catalysis emerged as a particularly powerful complement to Pd catalysis. The field of iridium-catalyzed allylic substitution reactions began to develop with the groundbreaking work of Takeuchi and Kashio, and subsequent research has revealed that iridium catalysts behave quite differently from their palladium counterparts [18]. The most notable distinction lies in their contrasting regioselectivity patterns. Palladium catalysts generally produce straight-chain products lacking chirality when reacting with monosubstituted allylic substrates, whereas iridium catalysts selectively generate branched products with high optical purity and precise control over the reaction site.

Furthermore, the development of chiral phosphoramidite ligands significantly advanced this field, with contributions from numerous research groups including Hartwig, Helmchen, Carreira, Alexakis, and You [19].

In general, soft nucleophiles that typically possess conjugate acids with p*K*_a_ values less than 25 have been utilized in most Pd and Ir-catalyzed allylic substitution reactions [20]. To address these limitations, copper-catalyzed processes have emerged as a promising alternative. Copper-catalyzed allylic substitutions are distinguished by their unique inner-sphere mechanistic pathway, which enables the incorporation of hard, non-stabilized nucleophiles **5** that have conjugate acids with p*K*_a_ values greater than 25 such as organolithium, organomagnesium, organozinc, and organozirconium reagents. This crucial distinction effectively expanded the scope of allylic substitution reactions beyond traditional boundaries.

The evolution of copper-catalyzed asymmetric allylic alkylation (AAA) has been remarkable since its initial development in 1995, when Bäckvall and van Koten first reported moderate enantioselectivity using Grignard reagents with allylic acetates [21–22]. This discovery triggered extensive research endeavors, significantly expanding the scope and efficiency of these reactions. A notable advancement came from Knochel's introduction of dialkylzinc reagents in 1999, which substantially broadened the range of applicable organometallic compounds [23].

Subsequent significant progress was achieved independently by Feringa and Alexakis through their exploration of phosphoramidite ligands with various organometallic nucleophiles [24–25]. The field was further advanced by the research group of Hoveyda, who made substantial contributions by introducing bidentate N-heterocyclic carbene (NHC)-based chiral ligands, achieving high selectivity with dialkylzinc reagents as nucleophiles [26]. They subsequently expanded the methodology by successfully employing triorganoaluminum reagents [27].

Recent developments in transition-metal-catalyzed AAA have increasingly turned to organoboron compounds [28–31]. These reagents offer numerous advantages, including non-toxicity, bench-stability, structural diversity, straightforward preparation, and broad commercial availability. Significant contributions include Sawamura's work with alkyl–9-BBN [32–37] as a nucleophile. These developments have collectively transformed copper-catalyzed AAA into a powerful and versatile tool in asymmetric synthesis, capable of employing a wide array of organometallic reagents that furnish the desired compounds with high efficiency and selectivity.

**Scheme 2:**
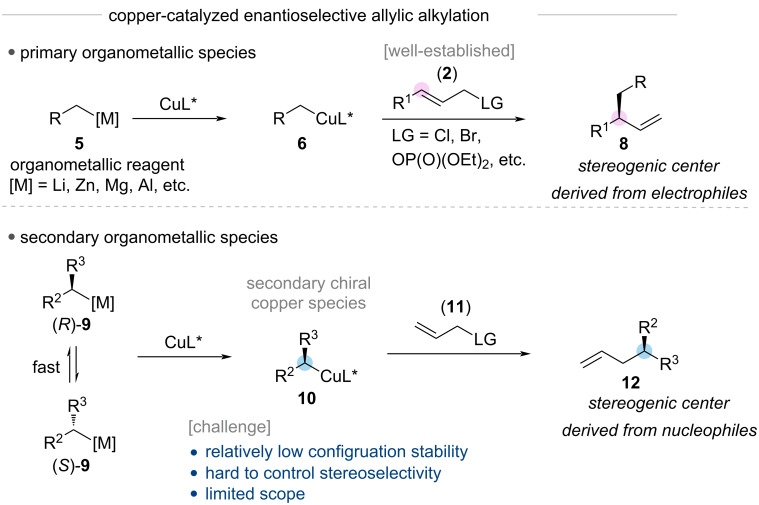
Formation of stereogenic centers in copper-catalyzed allylic alkylation reactions.

The regioselective asymmetric construction of stereogenic carbon centers from prochiral allylic substrates largely depends on the choice of the nucleophilic organometallic species (Scheme 2). For example, the asymmetric copper-catalyzed allylic alkylation utilizing organometallic species **5** bearing a primary carbon–metal bond predominantly constructs the stereogenic center derived from electrophiles. The stereoselective allylic substitution reaction with organometallic species **9** bearing a secondary carbon–metal bond has rarely been reported, despite its potential to enable complementary formation of the stereogenic center derived from nucleophiles. These reactions face significant challenges due to the relatively low configurational stability of the chiral secondary organometallic **9** and organocopper species **10** [38]. Therefore, the development of a more broadly applicable catalytic system that could accomplish copper-catalyzed stereoselective allylic alkylation with chiral secondary nucleophiles represents a crucial advancement in this field. In particular, a key objective is developing regio-, diastereo-, and enantioselective allylic substitution reactions that can effectively construct enantioenriched stereogenic centers from either allylic electrophiles or organometallic nucleophiles [39–40]. This advancement requires establishing catalytic systems that effectively utilize chiral secondary organocopper species, making the understanding of their nature and behavior crucial for expanding the synthetic utility of this transformation.

The purpose of this review is to present recent procedures for generating configurationally unstable organocopper species with secondary carbon–metal bonds, their unique properties, and related mechanistic insights. This review also aims to outline future research directions and prospects, contributing to the development of more efficient and selective copper-catalyzed AAA methodologies.

## Review

### Copper-catalyzed stereospecific coupling of chiral organometallic species with allylic electrophiles

The asymmetric construction of carbon–carbon bonds through copper-catalyzed AAA has emerged as a powerful synthetic tool in organic chemistry [41–45]. The generation of chiral secondary alkylcopper species has been a significant challenge in organic synthesis, primarily due to their inherent instability and tendency to racemization [38]. For generating the key chiral organocopper intermediates, two distinct approaches have been developed: one utilizing chiral organolithium species and the other employing chiral organoboron compounds.

#### Copper-mediated stereospecific coupling of chiral organolithium species with allylic electrophiles

**Scheme 3:**
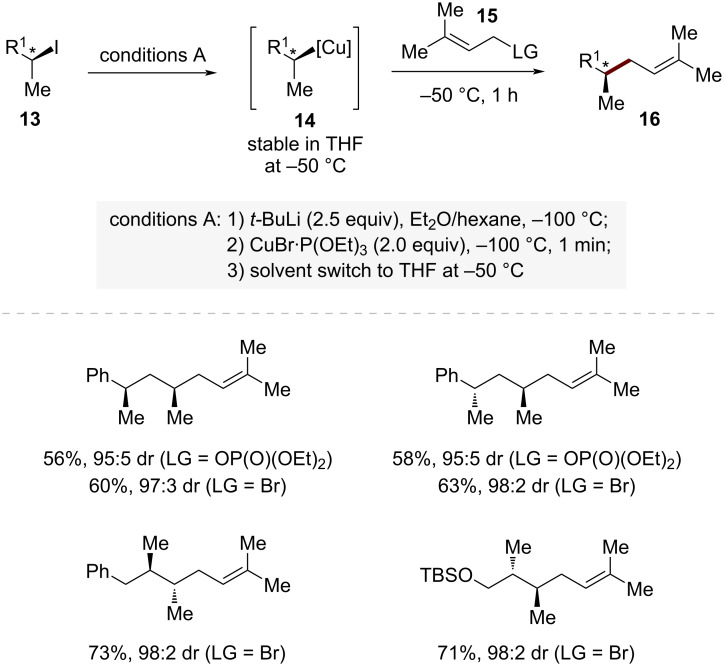
Copper-mediated, stereospecific S_N_2-selective allylic substitution through retentive transmetalation sequence.

In the organolithium approach, a breakthrough was achieved by Knochel and co-workers through carefully controlled reaction conditions (Scheme 3) [46]. Their methodology involves a stereoretentive I/Li exchange at −100 °C, followed by transmetalation with CuBr·P(OEt)_3_ to generate the secondary alkylcopper species **14**.

These organocopper species demonstrated remarkable reactivity in S_N_2-type additions to allylic bromides with exceptional regioselectivity (S_N_2/S_N_2' = >99:1). The reaction with 3-methylbut-2-en-1-yl bromide (**15**) was particularly noteworthy, as it exhibited superior selectivity compared to the corresponding phosphates. The high efficiency of this protocol was demonstrated through the synthesis of various functionalized alkenes with complete stereocontrol. For example, the reaction of *syn*-alkylcopper species **14** with 3-methylbut-2-en-1-yl bromide (**15**) provided the corresponding S_N_2 product **16** in excellent yield and stereoselectivity (S_N_2/S_N_2' = >99:1, dr = 97:3).

**Scheme 4:**
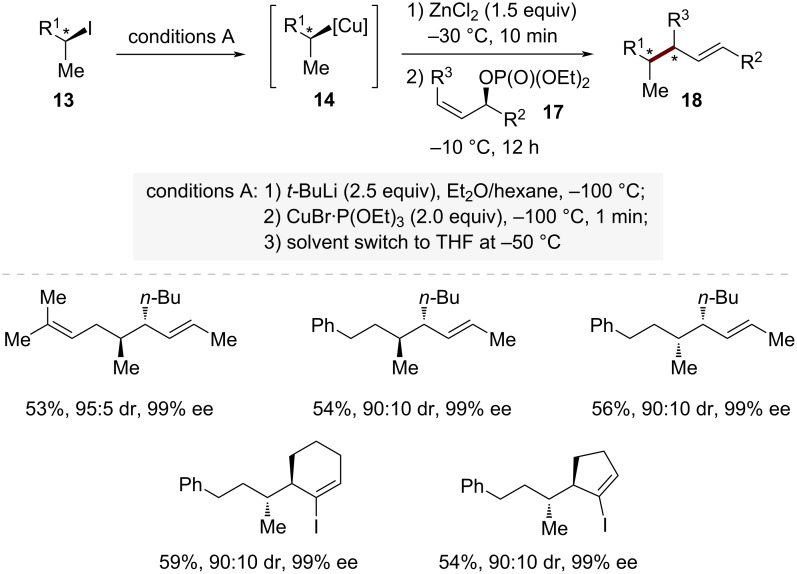
ZnCl_2_-promoted stereospecific S_N_2' allylic substitution of secondary alkylcopper species via sequential iodide–lithium–copper transmetalation.

Remarkably, the regioselectivity of these reactions could be completely reversed by adding zinc halides (Scheme 4). When treated with allylic phosphates **17** in the presence of ZnCl₂, these copper reagents **14** showed a dramatic shift in selectivity, favoring S_N_2' substitution. This exceptional reversed regioselectivity likely occurs through the in situ formation of copper–zinc mixed species [RCu·ZnX_2_·L] {X = Br, Cl; L = P(OEt)_3_}. Under these modified conditions, the reaction with allylic phosphates **17** proceeded with excellent S_N_2' selectivity (S_N_2/S_N_2' = 5:95) while maintaining high stereochemical fidelity. The versatility of this approach was further demonstrated through reactions with various chiral cycloallylic phosphates **17**, which consistently yielded the corresponding S_N_2' products **18** with high stereoselectivity.

The synthetic utility and broad applicability of this methodology was prominently demonstrated through the total synthesis of biologically important natural products. The high stereochemical selectivity of both S_N_2 and S_N_2' pathways enabled the efficient construction of complex molecular frameworks. Notably, this approach facilitated the enantioselective synthesis of three ant pheromones: (+)-lasiol, (+)-13-norfaranal, and (+)-faranal. The synthesis of (+)-lasiol was achieved through a highly selective S_N_2 substitution (S_N_2/S_N_2' = >99:1, dr = 98:2, 99% ee), while the preparation of (+)-13-norfaranal and (+)-faranal showcased the versatility of the methodology in constructing more complex terpene frameworks. These successful applications in natural product synthesis underscore the robustness and reliability of this copper-mediated transformation in creating stereochemically complex molecules.

Mechanistic investigations revealed several key factors controlling the stereochemical outcome of these transformations. The extremely low temperature (−100 °C) during the Li/I exchange is essential for preventing racemization of the configurationally labile organolithium intermediate. The subsequent transmetalation with CuBr·P(OEt)_3_ introduces P(OEt)_3_ as a supporting ligand, which plays a vital role in stabilizing the resulting chiral organocopper species **14**. A key breakthrough in this process was the discovery of a solvent effect: switching from Et_2_O/hexane to THF at −50 °C after organocopper species formation dramatically enhances configurational stability.

**Scheme 5:**
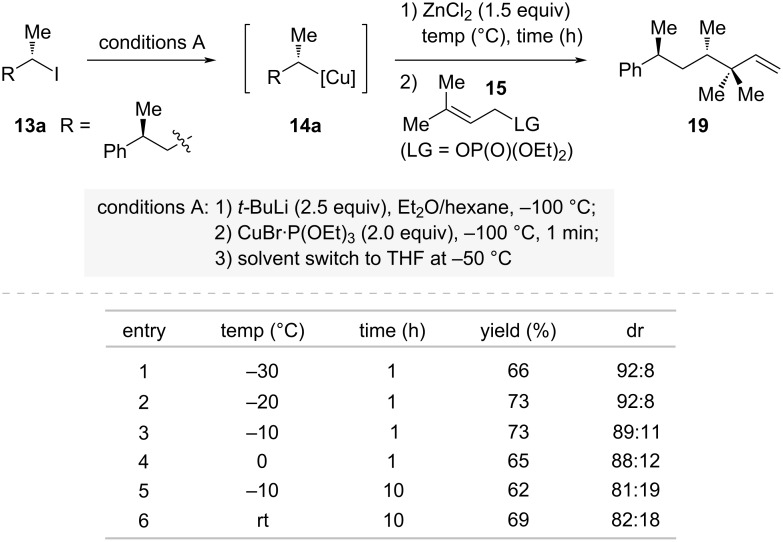
Temperature and time-dependent configurational stability of chiral secondary organocopper species.

Further investigations into the stability of the secondary organocopper species **14a** revealed the critical importance of reaction conditions after ZnCl_2_ addition (Scheme 5). When the reaction mixture was stirred at −30 °C for 1 hour (entry 1 in Scheme 5), the product **19** maintained a high diastereomeric ratio (dr), indicating minimal racemization. However, extending the stirring time or increasing the reaction temperature led to a significant decrease in dr values, suggesting partial racemization. This observation highlights the importance of careful experimental handling of [RCu·ZnX_2_·L] species {X = Br, Cl; L = P(OEt)_3_}, where both temperature control and reaction time must be precisely managed to maintain stereochemical integrity. Through these careful experimental controls, Knochel and co-workers effectively addressed the long-standing challenge of handling these traditionally unstable chiral organometallic intermediates.

#### Copper-catalyzed stereospecific coupling of chiral organoboron species with allylic electrophiles

**Scheme 6:**
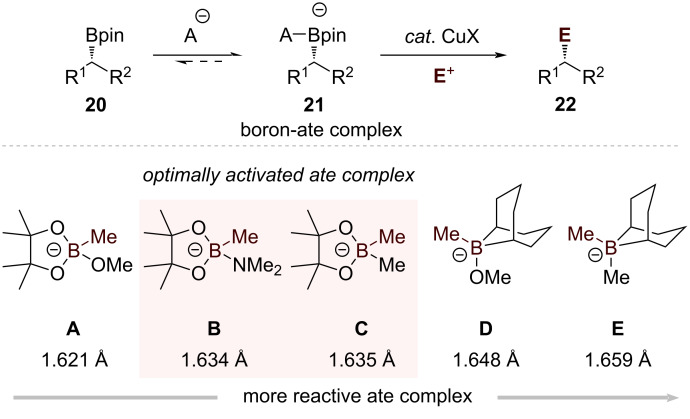
DFT analysis of B–C bond lengths in various boronate complexes and correlation with reactivity.

While the direct formation of chiral copper species from organolithium compounds provides an efficient route to stereospecific allylic alkylation products, the requirement of stoichiometric amounts of the copper reagent limits its practical application [46]. An alternative approach utilizing more configurationally stable organoboron compounds was recently developed by Morken and co-workers, which employs only catalytic amounts of copper [47–48]. Their strategy uses chiral secondary organoboron compounds **20** as precursors to generate chiral alkylcopper species through a carefully controlled activation and transmetalation sequence (Scheme 6).

**Scheme 7:**
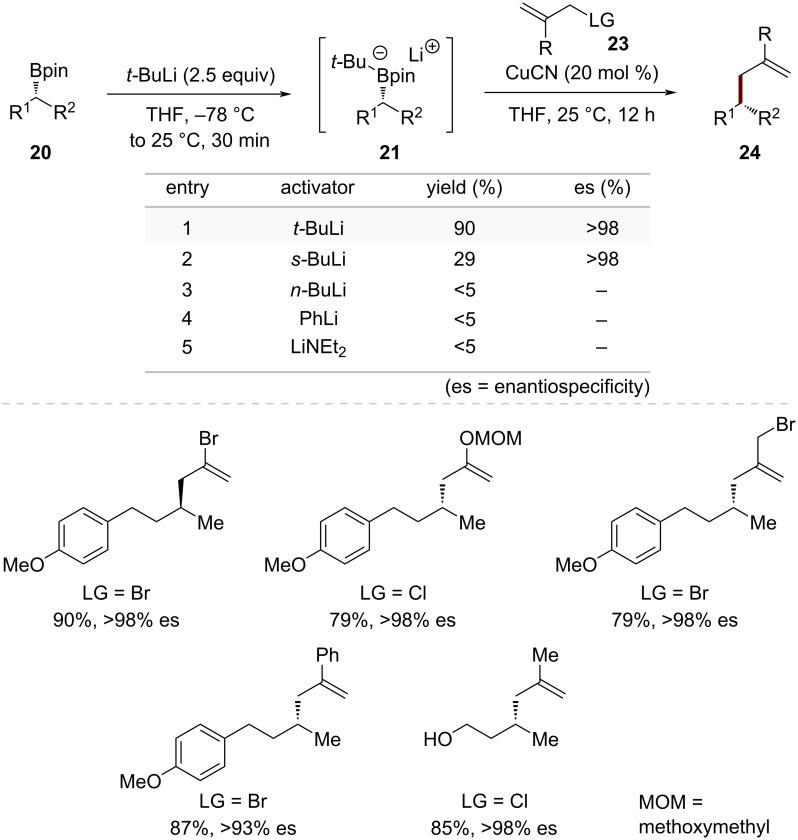
Copper-catalyzed stereospecific allylic alkylation of secondary alkylboronic esters via *tert*-butyllithium activation.

For secondary boronic esters, Morken and co-workers conducted a systematic investigation to develop an efficient activation strategy [47]. Computational studies using DFT revealed an important relationship between the length of the boron–carbon bonds and the corresponding complex's chemical behavior (Scheme 6). Among the analyzed four-coordinate boron species, the unreactive trialkoxyborate complex **A** exhibited the most compact B–C bond at 1.621 Å. Following a clear trend, the B–C bond distance progressively lengthened as oxygen atoms were substituted with elements of lower electronegativity, reaching 1.648 Å in the reactive alkoxytrialkyl complex **D** and 1.659 Å in the tetraalkyl "ate" complex **E**. The B–C bond lengths in amido- and alkyl-substituted boronic ester complexes **B** and **C** fell between these extremes, suggesting an intermediate level of activation. These findings prompted investigation into whether such moderate activation strategies could facilitate copper-mediated coupling reactions of sterically demanding alkylboronic esters under mild conditions. After thorough reaction optimization, *t-*BuLi emerged as the superior activating agent, outperforming other organolithium compounds including *s-*BuLi, *n-*BuLi, and PhLi. The optimized protocol, employing CuCN as a catalyst, enabled efficient coupling between *t-*BuLi-activated complexes **10** and allylic halides **11**, furnishing products **12** with complete retention of stereochemistry and high yields (Scheme 7). The method's versatility and consistent stereochemical outcome highlight its practical utility.

**Scheme 8:**
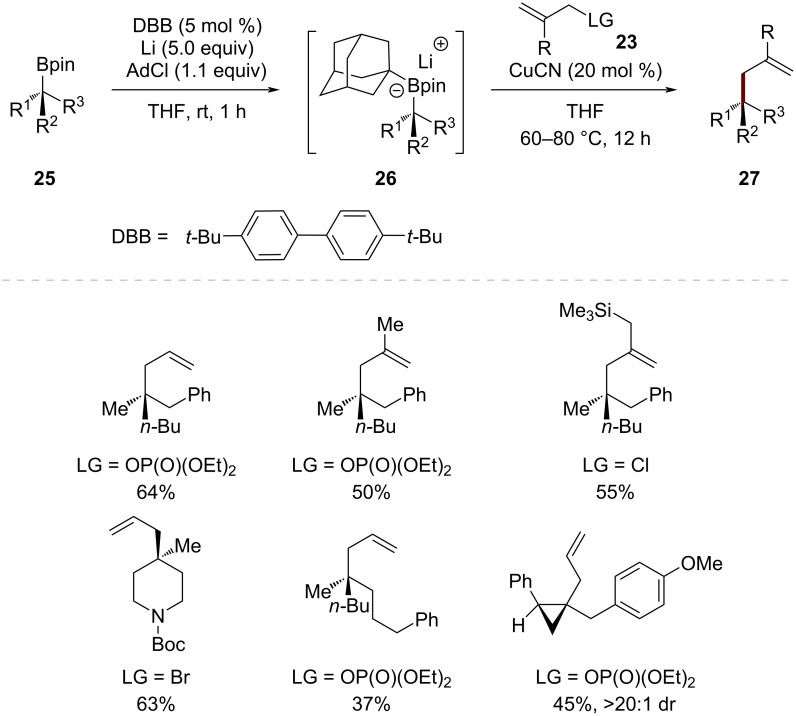
Copper-catalyzed stereospecific allylic alkylation of chiral tertiary alkylboronic esters via adamantyllithium activation.

Their subsequent work with chiral tertiary boronic esters **25** revealed an effective strategy for constructing quaternary stereogenic centers through allylic substitution reactions (Scheme 8) [48]. By employing in situ-generated adamantyllithium as an activator, they found that tertiary alkyl groups underwent selective transmetalation over the adamantyl group. Under optimized conditions, the activated chiral tertiary boronic esters **26** underwent efficient copper-catalyzed coupling with allylic electrophiles **23** to provide products **27** bearing quaternary stereogenic centers with high stereospecificity. This methodology represents a significant advancement in the construction of challenging all-carbon quaternary stereogenic centers through stereospecific allylic substitution reactions.

**Scheme 9:**
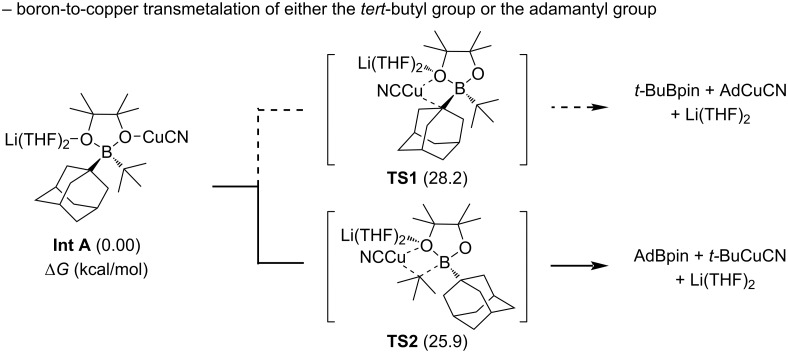
DFT-calculated energy surface for boron-to-copper transmetalation of either the *tert*-butyl group or the adamantyl group.

To elucidate the origin of the selective boron group transfer, X-ray crystallographic analysis of the (*tert*-butyl)(adamantyl)Bpin·Li(THF)_2_ complex revealed that the B–(adamantyl) bond is shorter than the B–(*tert*-butyl) bond (1.673 vs 1.692 Å). DFT calculations further illuminated the underlying mechanism by comparing two distinct transition states: **TS1** involving adamantyl transfer and **TS2** involving *tert*-butyl transfer (Scheme 9). Analysis of these transition states revealed that both require significant pyramidalization of the transferring carbon center, with the barrier for adamantyl transfer (**TS1**) being 2.3 kcal/mol higher than that for *tert*-butyl transfer (**TS2**). The structural features of the *tert*-butyl group allow more efficient pyramidalization compared to the rigid adamantyl framework, suggesting that the flexibility of the transferring group plays a crucial role in facilitating transmetalation.

### Copper hydride chemistry for enantioselective allylic substitution reactions

**Scheme 10:**
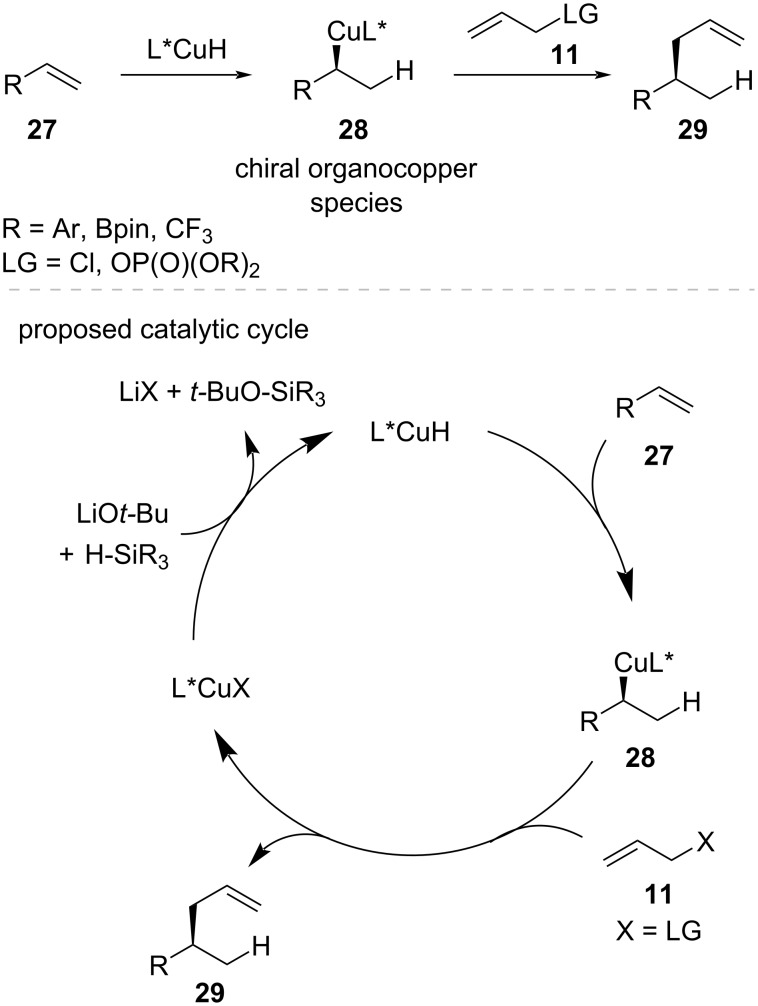
CuH-catalyzed enantioselective allylic substitution and postulated catalytic cycle.

Among the various approaches in copper-catalyzed asymmetric allylic substitution, copper hydride (CuH) catalysis has received significant attention due to its unique ability to generate configurationally well-defined chiral organocopper species **28** under mild conditions without requiring stoichiometric organometallic reagents [49] (Scheme 10). The distinctive reactivity of the CuH species allows for precise control over the stereochemical outcome through the regio- and enantioselective hydrocupration of olefins **27**, followed by stereospecific trapping with allylic electrophiles **11**.

#### Enantioselective hydroallylation and allylboration of styrenes

**Scheme 11:**
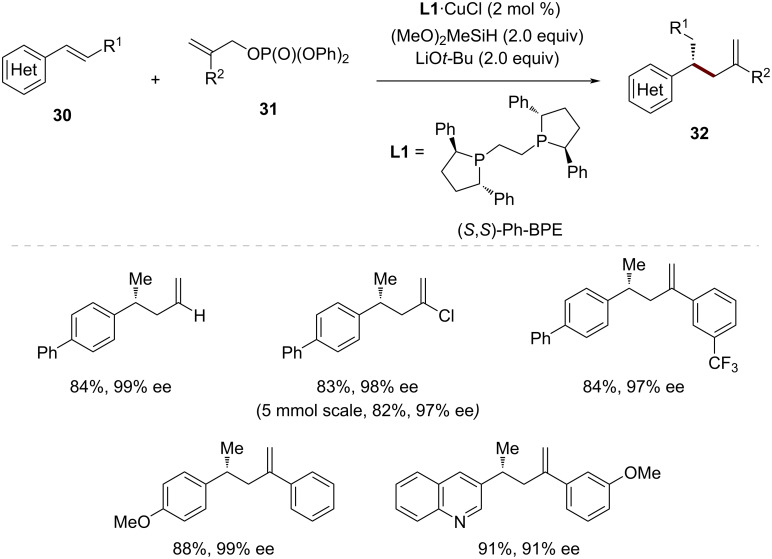
CuH-catalyzed enantioselective allylic substitution of vinylarenes.

A significant advance in the CuH-catalyzed enantioselective allylic substitution was reported by Buchwald and co-workers in 2016, who demonstrated the first successful hydroallylation of vinylarenes **30** using allylic phosphate electrophiles **31** (Scheme 11) [50]. This methodology is distinguished by its ability to efficiently construct configurationally well-defined stereogenic centers during C–C-bond formation through the intermediacy of benzylic copper species.

Initial investigations suggested that the reaction outcome was highly dependent on both the leaving group of the allylic electrophile and the choice of the supporting ligand. When (+)-1,2-bis{(2*S*,5*S*)-2,5-diphenylphospholano}ethane {(*S*,*S*)-Ph-BPE} (**L1**) was employed as the supporting chiral ligand, initially allylic chloride was found to provide the desired product **32** with excellent enantioselectivity, although in moderate yield. Notably, the presence of a chloride anion in the reaction mixture proved crucial for high enantioselectivity, leading to the discovery that a 1:1 complex of copper(I) chloride and (*S*,*S*)-Ph-BPE (**L1**) could serve as an optimal catalyst system. Further optimization revealed LiO*t-*Bu and diphenyl phosphate as the optimal metal alkoxide and leaving group, delivering the desired product **32** in high yield and high enantioselectivity at room temperature with only 2 mol % catalyst loading.

The scope of this transformation proved to be remarkably broad. In addition to the parent allyl group, a variety of 2-substituted electrophiles **31** could be applied. These included those bearing alkyl groups of varying steric demand, halides, and both electron-rich and electron-poor aryl substituents. The olefin coupling partner scope was equally impressive, tolerating styrenes with diverse electronic properties as well as those containing sensitive functional groups such as esters, amides, and vinyl halides, to yield the desired β-chiral olefins in high enantioselectivity. Notably, the methodology could even be applied to vinylferrocene and vinylsilane derivatives, providing rapid access to highly enantioenriched organometallic and organosilicon compounds.

Mechanistic studies using a deuterium-labeled allylic phosphate revealed that the C–C-bond formation occurs through an S_N_2'-like process, with attack of the organocopper species at the 3-position of the allylic phosphate. The absolute stereochemistry of the products was found to be consistent with that of previously reported CuH-catalyzed transformations using (*S*,*S*)-Ph-BPE (**L1**) as the supporting ligand, suggesting a common mode of stereoinduction.

In parallel, Hoveyda and co-workers demonstrated the first copper-catalyzed enantioselective allylic substitution of styrenes utilizing Cu–Bpin species [51]. Through implementation of a chiral NHC–Cu complex with B_2_(pin)_2_, they achieved the highly selective formation of homoallylic boronic esters with excellent enantioselectivities (up to 98% ee). This methodology represents a complementary approach to the hydroallylation protocol developed by the research group of Buchwald, enabling a direct construction of versatile organoboron compounds that can be readily elaborated to more complex molecular architectures.

#### Enantioselective hydroallylation of vinylboronic esters

**Scheme 12:**
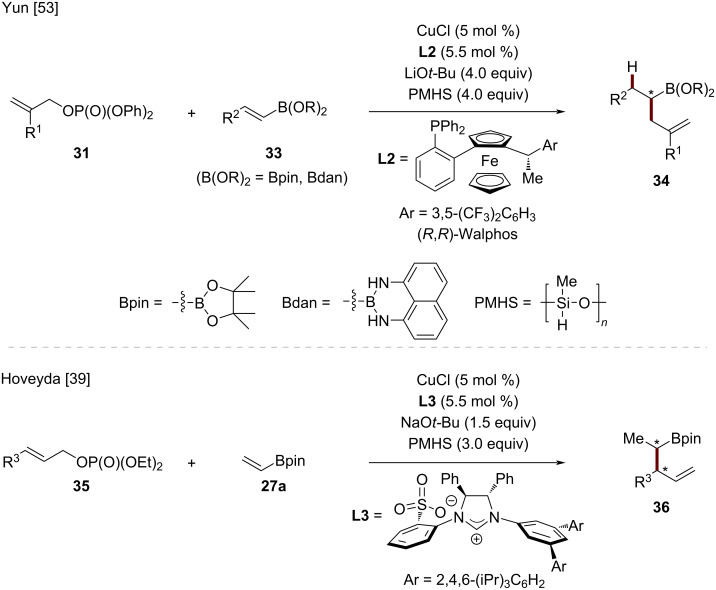
CuH-catalyzed stereoselective allylic substitution of vinylboronic esters.

Following the initial demonstration by Buchwald that styrenes are viable substrates for the CuH-catalyzed hydroallylation, the development of methods applicable to vinylboronic esters presented unique opportunities for the synthesis of versatile chiral organoboron compounds [52]. A breakthrough in this field was achieved in 2016 by Yun and co-workers, who developed a copper-catalyzed regio- and enantioselective hydroallylation of vinylboronic acid pinacol esters (Bpin) and 1,8-diaminonaphthalene boramides (Bdan) **33** (Scheme 12) [53]. Subsequently, Hoveyda and co-workers introduced a complementary approach focused on the diastereoselective formation of homoallylic boronic esters **36** through a carefully controlled sequence of hydrocupration and allylic substitution [39].

Through optimization studies, Yun found that CuCl with the Walphos ligand (**L2**) in Et_2_O provided optimal results, delivering the desired products with up to 99% ee. This methodology worked effectively with both pinacol boronic esters (Bpin) and 1,8-diaminonaphthalene boramides (Bdan), showing broad vinylboron substrate scope across alkyl, aryl, and heteroaryl substituents. The practical utility of the enantioenriched alkylboronic esters **34** was demonstrated through the efficient synthesis of (*S*)-massoialactone.

Subsequently, Hoveyda developed a highly selective copper-catalyzed allylic substitution of (*E*)-1,2-disubstituted allylic phosphates **35** with vinylBpin **27a** using polymethylhydrosiloxane (PMHS) as the hydride source [39]. The reaction, catalyzed by a sulfonate-containing chiral NHC–Cu complex, proceeded with excellent chemo-, regio- (S_N_2'-), diastereo-, and enantioselectivity to afford homoallylic boronates **36**. The resulting organoboron compounds could be oxidized to secondary homoallylic alcohols, providing an alternative to traditional crotyl addition to acetaldehyde. This methodology represents the first example of highly enantio- and diastereoselective copper-catalyzed allylic substitution that controls vicinal stereogenic centers.

**Scheme 13:**
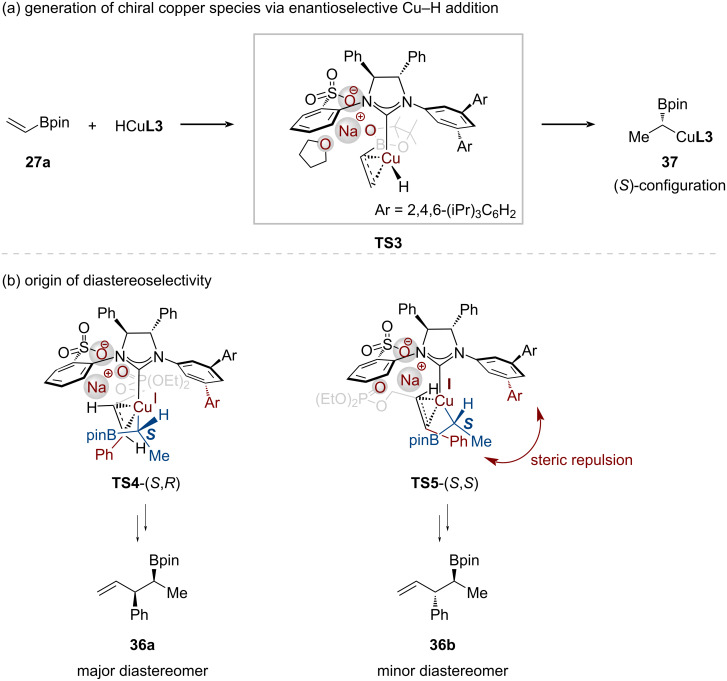
(a) Generation of chiral copper species via enantioselective CuH addition to vinylBpin. (b) Regarding the origin of diastereoselectivity in CuH-catalyzed enantioselective allylic substitution.

DFT calculations revealed that the high enantioselectivity (98:2 er) originates from a face-selective formation of the chiral organocopper species **37** (Scheme 13a). The 3,5-(2,4,6-triisopropylphenyl) substituent of the NHC ligand **L3** effectively blocks one face of the reactive center, forcing vinylBpin **27a** to approach the CuH complex from the less hindered face. This precise steric control during copper complex formation and subsequent selective olefin insertion results in the high levels of enantioselectivity (98:2 er) observed experimentally.

DFT calculations further elucidated the origin of the high diastereoselectivity (up to 96:4 dr) in the allylic substitution step (Scheme 13b). Analysis of the competing transition states showed that the chiral α-borylalkylcopper species **37** could approach the olefinic moiety of allylic electrophile **35** from either the *re*- or *si*-face. The *re*-face approach is energetically favored, minimizing steric interactions between the bulky aryl substituent of NHC ligand **L3** and the allylic electrophile **35**. In contrast, a *si*-face attack leads to a higher-energy transition state due to significant steric repulsion.

#### Enantioselective hydroallylation of vinyltrifluoromethyl compounds

**Scheme 14:**
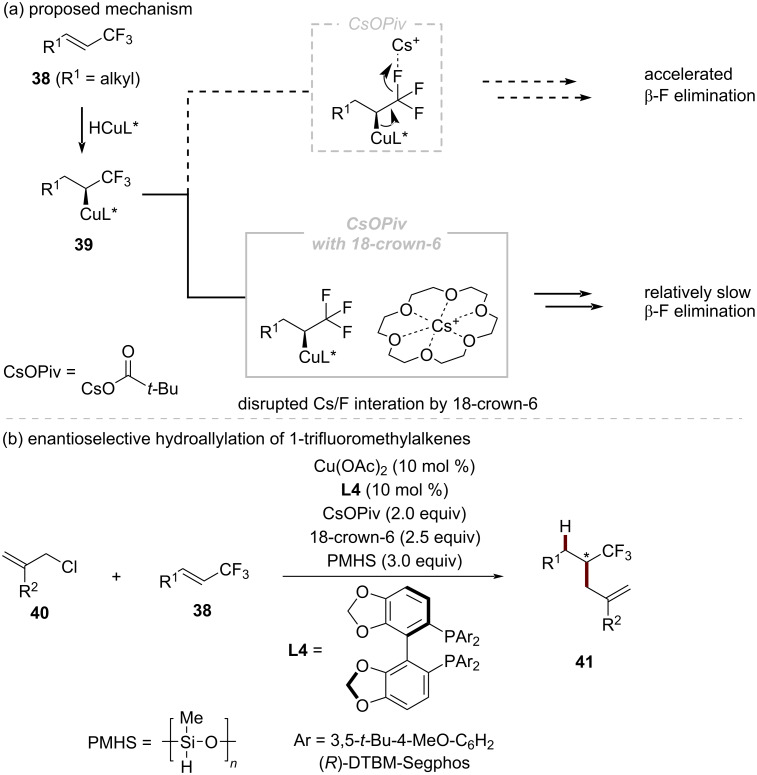
CuH-catalyzed enantioselective allylic substitution of 1‐trifluoromethylalkenes with 18-crown-6.

The direct functionalization of 1-trifluoromethylalkenes **38** through copper catalysis has been challenging due to the tendency of α-CF_3_-substituted alkylcopper intermediates **39** to undergo undesired β-F elimination (Scheme 14a) [54]. In 2021, Hirano and co-workers made a significant advance in this area by disclosing a copper-catalyzed regio- and enantioselective hydroallylation of 1-trifluoromethylalkenes **38** with hydrosilanes and allylic chlorides **40** (Scheme 14b) [55]. In their work, a chiral α-CF_3_ alkylcopper intermediate **39** was formed through the regio- and enantioselective hydrocupration of electron-deficient alkenes **38** with an in situ-generated CuH species. Subsequent electrophilic trapping of the α-CF_3_-alkylcopper species **39** with the allylic electrophile **40** leads to the optically active hydroallylated product **41**. The key to the success of this protocol was the combination of an appropriate chiral bisphosphine ligand, (*R*)-DTBM-Segphos (**L4**), and the use of 18-crown-6 to suppress the otherwise predominant β-F elimination from the α-CF_3_-alkylcopper intermediate **39**. Detailed kinetic studies confirmed the effect of the crown ether. When KOPiv was employed alone, the initial rate of defluorination increased by 1.57-fold, whereas the addition of 18-crown-6 reduced this acceleration to 1.22-fold. These observations supported the hypothesis that the crown ether effectively disrupts the interaction between the alkali metal cation and fluorine atom, thereby decreasing the rate of β-F elimination.

This asymmetric copper-catalyzed protocol represents one of the rare examples that allows to construct non-benzylic and non-allylic CF_3_-substituted C(sp^3^) stereogenic centers. The synthetic utility of this allylation process was demonstrated through the facile functionalization of the allylic moiety in the enantioenriched product **41**, providing access to optically active CF_3_-containing compounds bearing various functionalities. Notably, no erosion of enantiomeric excess was observed during any of the transformations.

#### Double CuH insertion into alkynes for regiodivergent allylic substitution

Generating chiral secondary alkylcopper species in situ through sequential hydrocupration of terminal alkynes in a chemo-, regio-, and enantioselective manner represents a recent advance in copper hydride chemistry. Along these lines, in 2024, Su and co-workers developed a strategy for the copper-catalyzed regio- and enantioselective synthesis of secondary homoallylboron compounds by assembling four readily available starting materials: terminal alkynes, HBdan, polymethylhydrosiloxane (PMHS), and allylic phosphates, through a complex cascade hydroboration and hydroallylation sequence [56].

**Scheme 15:**
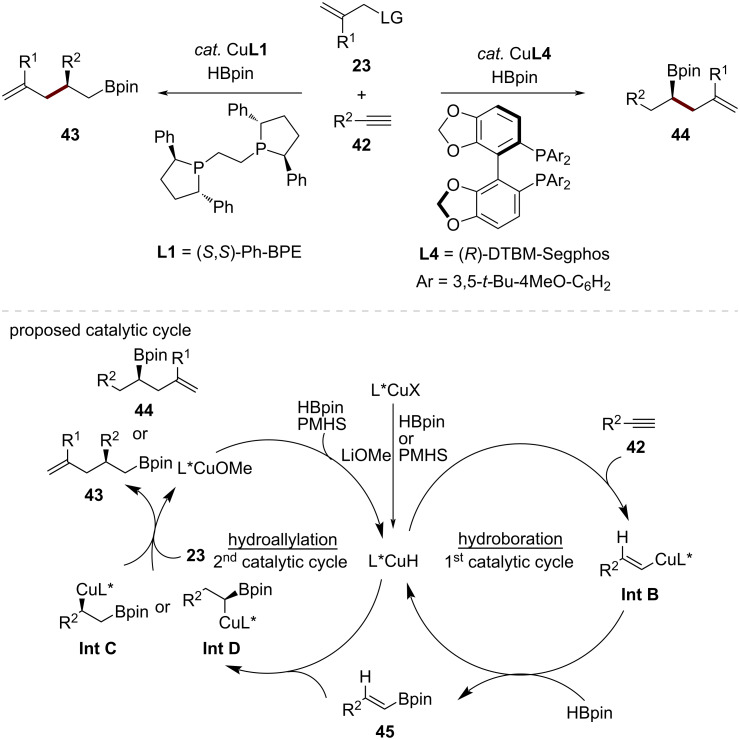
CuH-catalyzed enantioselective allylic substitution of terminal alkynes.

Shortly after this work, Xiong, Zhu, and co-workers reported a ligand-controlled copper-catalyzed regiodivergent asymmetric difunctionalization of terminal alkynes through a cascade process involving initial hydroboration followed by a hydroallylation (Scheme 15) [57]. Employing a catalytic system consisting of (*R*)-DTBM-Segphos (**L4**) and CuBr resulted in the exclusive 1,1-difunctionalization of aryl- and alkyl-substituted terminal alkynes **42**, including the industrially relevant acetylene and propyne. Interestingly, switching to the ligand (*S*,*S*)-Ph-BPE (**L1**) resulted in the asymmetric 1,2-difunctionalization of aryl-substituted terminal alkynes **42**. The high levels of regio- and stereoselectivity achieved under mild conditions render this method attractive for constructing complex chiral molecular architectures.

A plausible mechanistic pathway for this cascade asymmetric hydroboration and hydroallylation of alkynes was proposed based on a series of control experiments, including deuterium-labeling experiments and DFT calculations. The first hydroboration catalytic cycle is initiated by L*CuH species (L* = a chiral ligand) formed in situ through the combination of CuBr, LiOMe, and HBpin in the presence of a chiral ligand. Subsequent alkyne migratory insertion provides a vinyl cuprate intermediate **Int B**, followed by σ-bond metathesis with HBpin to afford a vinylboronic ester intermediate **45** alongside the regenerated L*CuH catalyst, completing the first catalytic cycle. Subsequently, a ligand-controlled regioselective migratory insertion of L*CuH into the vinylboronic ester **18** delivers the corresponding chiral alkylcopper species **Int C** or **Int D**, which undergoes an S_N_2'-like pathway with allylic phosphates **23** to generate the chiral products **43** or **44** along with the release of L*CuOR species. A σ-bond metathesis of this alkoxycopper species with HBpin and/or PMHS regenerates the L*CuH catalyst, completing the secondary asymmetric regiodivergent hydroallylation cycle.

### Copper-catalyzed enantiotopic-group-selective allylation of 1,1-diborylalkanes

The generation of chiral non-racemic organocopper species through enantiotopic-group-selective transmetalation of 1,1-diborylalkanes **47** has recently garnered significant interest [58]. This methodology has emerged as a powerful strategy for constructing stereogenic centers with high levels of stereocontrol, offering important complementarity to established CuH-catalyzed processes. While CuH-catalyzed processes have proven highly effective for numerous substrates, they exhibit inherent limitations with molecules possessing unsaturated functionalities due to competitive hydrocupration pathways. The approach using 1,1-diborylalkanes **47** circumvents these chemoselectivity issues and enables a selective allylic substitution of substrates containing olefins and alkynes with excellent stereoselectivity. Importantly, this orthogonal reactivity complements established CuH-catalyzed procedures, significantly expanding the scope of copper-catalyzed asymmetric allylic substitution reactions.

**Scheme 16:**
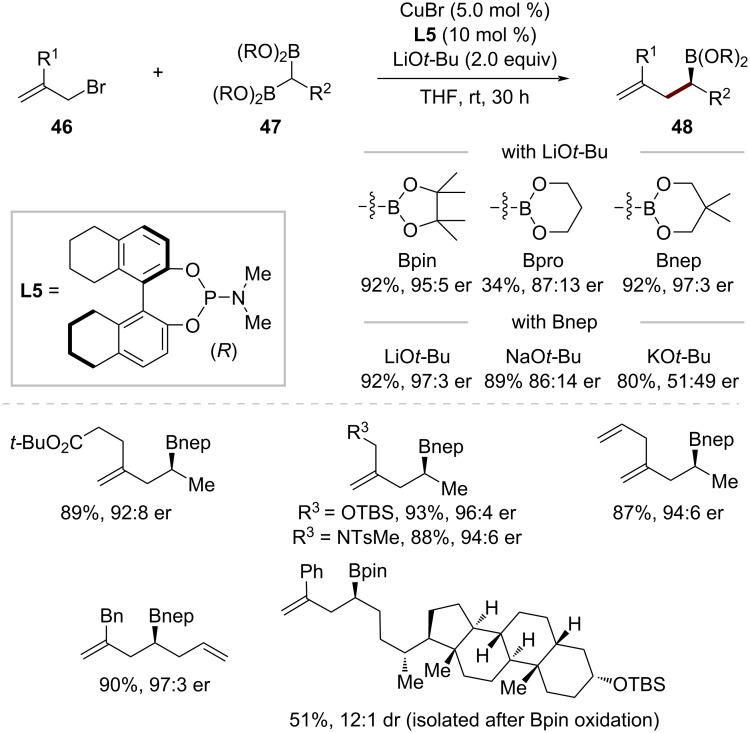
Copper-catalyzed enantiotopic-group-selective allylic substitution of 1,1-diborylalkanes.

In 2021, Cho, Baik, and co-workers reported the first enantioselective copper-catalyzed allylation of 1,1-diborylalkanes **47** using an H_8_-BINOL-derived phosphoramidite ligand **L5**, achieving exceptional enantiocontrol (Scheme 16) [59]. Their studies revealed the critical role of both the alkali metal cation and boronic ester moiety. While LiO*t-*Bu provided excellent enantioselectivity (er = 95:5), NaO*t-*Bu and KO*t-*Bu performed poorly due to competitive transition-metal-free processes via α-borylcarbanion formation. The substituent of the boron atom proved equally important: neopentylglycolato groups (Bnep) outperformed pinacolato (Bpin) or propanediolato groups (Bpro) in stereoselectivity. The optimized conditions showed a broad scope, tolerating both 1,1-diborylalkanes with *N*-tosyl-protected amines and TBS-protected alcohols, as well as substrates containing alkenes and alkynes. Various allylic bromides **46** with electron-rich and electron-deficient aryl substituents worked well, giving homoallylic boronic esters **48** that contain a boron-substituted stereogenic center derived from the prochiral 1,1-diborylalkanes **47** in good yields with high stereoselectivity.

**Scheme 17:**
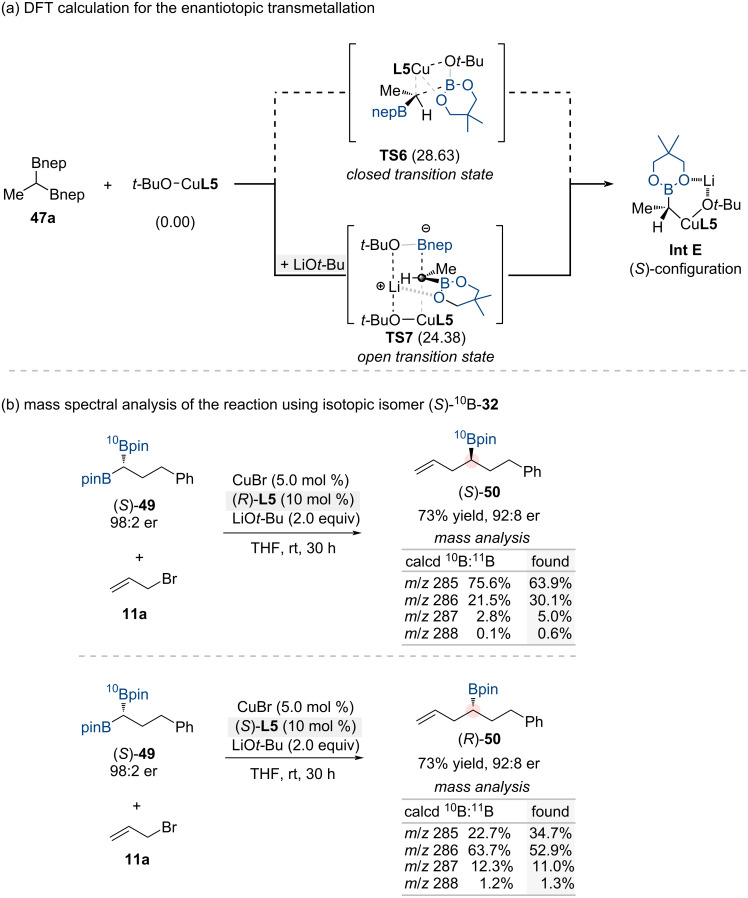
(a) Computational and (b) experimental studies to elucidate the mechanistic details of the enantiotopic-group-selective transmetalation.

DFT calculations of the enantiotopic-group-selective transmetalation between 1,1-diborylalkanes and chiral copper species revealed two possible transition states: an open transition state **TS7** facilitated by LiO*t*-Bu and a closed transition state **TS6** without base assistance (Scheme 17a). The significant energy difference between these pathways (ΔΔ*G*^‡^ = 4.3 kcal/mol) strongly favors the open transition state **TS7** mechanism, attributed to reduced steric interactions and enhanced electronic stabilization through lithium coordination. This explains the critical role of lithium in achieving a high enantioselectivity. Isotope-labeling experiments using ^10^B-enriched 1,1-diborylalkanes (*S*)-**49** further supported this mechanism, showing a stereoinvertive transmetalation between the enriched substrate and chiral copper species, consistent with the calculations (Scheme 17b).

**Scheme 18:**
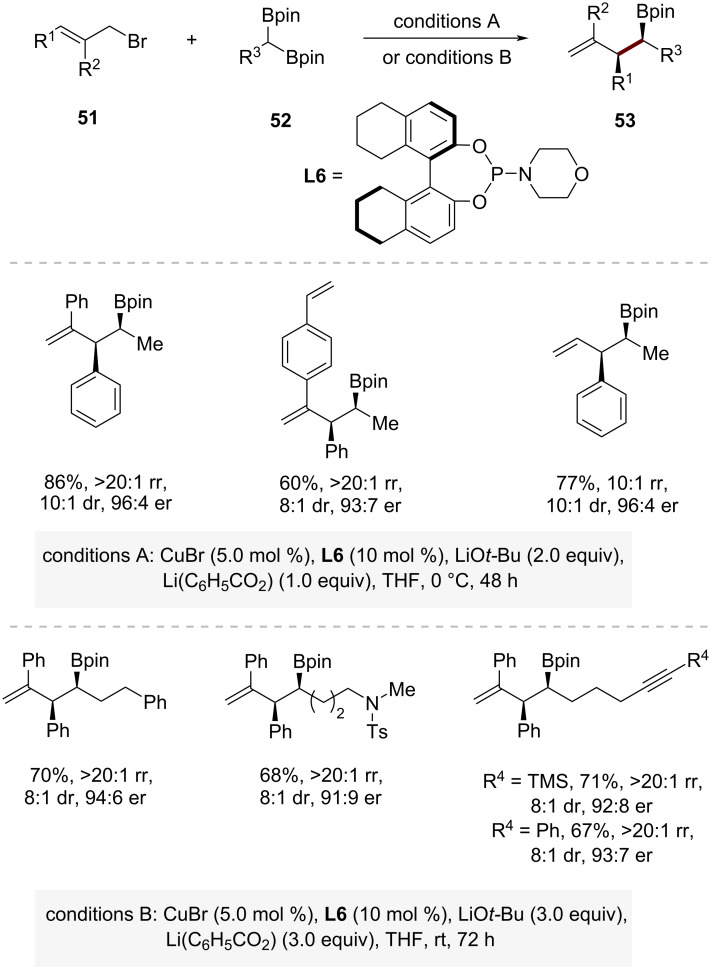
Copper-catalyzed regio-, diastereo- and enantioselective allylic substitution of 1,1-diborylalkanes.

In 2024, Cho and co-workers have developed a more stereoselective approach that can significantly broaden the scope of accessible electrophiles using 1,1-diborylalkanes **52** as pronucleophiles (Scheme 18) [40]. The asymmetric copper-catalyzed allylic alkylation enabled the efficient coupling of 1,1-diborylalkanes **52** with various allylic bromides **51**, achieving high levels of regio-, diastereo-, and enantioselectivity (rr = >20:1, dr = >8:1, up to 96:4 er). Under slightly modified conditions, a wide range of 1,1-diborylalkanes bearing an *N*-tosyl-protected amine as well as alkene and alkyne moieties underwent efficient coupling with allylic bromides. A notable advantage of this synthetic approach is that it provides a distinct alternative to traditional CuH-catalyzed allylic alkylation reactions. The method shows exceptional compatibility with 1,1-diborylalkanes bearing unsaturated functional groups such as alkenes or alkynes.

**Scheme 19:**
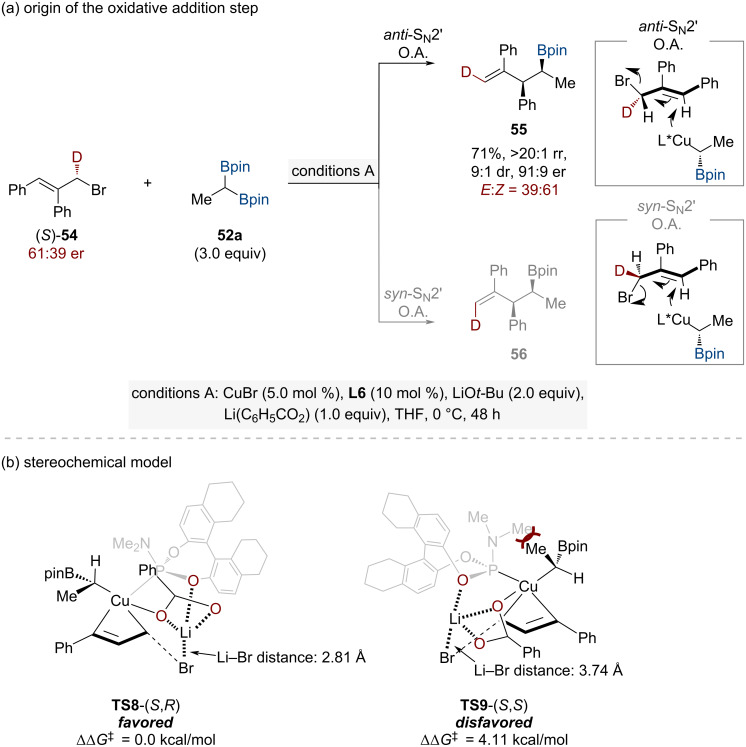
(a) Experimental and (b) computational studies to understand the stereoselectivities in oxidative addition step.

The copper-catalyzed asymmetric allylic substitution occurs via two possible pathways: *anti*-S_N_2' and *syn*-S_N_2' oxidative addition. To determine which pathway is operative, deuterium-labeling studies were conducted using an enantioenriched, isotopically labeled allylic bromide (*S*)-**54** (61:39 er) and 1,1-diborylethane **52a** under optimized reaction conditions (Scheme 19a). The resulting product **55** formed with 39:61 *E/Z* selectivity, indicating an *anti*-S_N_2' oxidative addition mechanism. In this pathway, the chiral α-borylalkylcopper intermediate approaches from the face opposite to the leaving group, consistent with the observed stereochemical outcome.

To explore the stereochemical origins and examine the mechanistic influence of the lithium benzoate additive, computational density functional theory calculations were performed focusing on the oxidative addition transition states (*S*,*R*)-**TS8** and (*S*,*S*)-**TS9** (Scheme 19b). The theoretical analysis revealed a notable energy difference between these diastereomeric transition states, with (*S*,*R*)-**TS8** being 4.11 kcal/mol lower in energy compared to (*S*,*S*)-**TS9**. Both transition states demonstrated a lithium center's coordination involving bromide, benzoate, and ligand oxygen atoms. The (*S*,*R*)-**TS8** transition state exhibited a significantly shorter Li–Br interaction distance (2.81 Å compared to 3.74 Å in (*S*,*S*)-**TS9**), offering mechanistic insight into the observed stereochemical outcome.

## Conclusion

This review highlights the evolution of strategies for generating and utilizing chiral nonracemic organocopper species with secondary carbon–metal bonds in asymmetric allylic substitution reactions. Early approaches relied on the stereospecific transmetalation of configurationally stable organometallic reagents, initially employing stoichiometric copper salts with chiral secondary organolithium species. The field then advanced through the development of more practical systems using configurationally stable chiral organoboron compounds, which enabled catalytic copper processes through carefully controlled activation.

Despite significant progress in copper hydride (CuH) catalysis eliminating the need for stoichiometric organometallic reagents, the scope of viable substrates remains restricted. While this methodology works effectively with olefins bearing electronic directing groups (such as aryl, boryl, or trifluoromethyl substituents) that guide regioselective hydrocupration, unactivated alkyl-substituted alkenes pose a persistent challenge. The recent breakthrough in CuH-catalyzed enantioselective and diastereoselective allylic alkylation of vinylboronic esters underscores both the current limitations and the potential for expanding substrate compatibility in this field.

Most recently, a complementary approach utilizing enantiotopic-group-selective transmetalation of 1,1-diborylalkanes has emerged. This method enables efficient coupling with various allylic electrophiles while achieving high levels of regio-, diastereo-, and enantioselectivity. Significantly, this strategy offers unique compatibility with substrates containing unsaturated functional groups such as alkenes or alkynes, overcoming a key limitation of conventional CuH catalysis.

Looking forward, several opportunities exist for further development in this field. The configurational stability of secondary alkylcopper species suggests broader applications in stereoselective transformations, particularly in the strategic construction of vicinal stereogenic centers through copper-catalyzed asymmetric allylic substitution reactions. While current methodologies have predominantly focused on generating single stereogenic centers, the rational design of compatible allylic electrophiles would provide an intriguing opportunity for accessing diverse stereochemical arrangements. The ability to precisely control multiple contiguous stereogenic centers would significantly expand the synthetic utility of such transformations, particularly in the synthesis of complex molecules such as natural products and pharmaceuticals [60]. Furthermore, future efforts could also focus on developing new catalyst systems for the copper-catalyzed stereoselective C–C-bond formation, as the mechanistic understanding gained from related studies may inform ligand design and expand the scope of nucleophiles and allylic electrophiles in this field. The continued development of these methodologies will be crucial for advancing the field of asymmetric synthesis.

## Data Availability

Data sharing is not applicable as no new data was generated or analyzed in this study
